# P-725. Predictive Diagnostic of Mpox Using Machine Learning Model with Clinical Information

**DOI:** 10.1093/ofid/ofaf695.936

**Published:** 2026-01-11

**Authors:** Masahiro Ishikane, Raiki Yoshimura, Noriko Iwamoto, Michiyo Suzuki, Noriko Fuwa, Yuichiro Nagase, Takeru Matsuura, Ryoko Asari, Natsuko Kaku, Yasutoshi Kido, Shingo Iwami, Norio Ohmagari

**Affiliations:** National Center for Global Health and Medicine, Japan Institute for Health Security (JIHS), Shinjuku, Tokyo, Japan; Nagoya University, Nagoya-city, Aichi, Japan; Japan Institute for Health Security, Shinjuku-ku, Tokyo, Japan; Japan Institute for Health Security, Shinjuku-ku, Tokyo, Japan; Japan Institute for Health Security, Shinjuku-ku, Tokyo, Japan; Japan Institute for Health Security, Shinjuku-ku, Tokyo, Japan; Nagoya University, Nagoya-city, Aichi, Japan; Osaka Metropolitan University, Osaka-city, Osaka, Japan; Osaka Metropolitan University, Osaka-city, Osaka, Japan; Osaka Metropolitan University, Osaka-city, Osaka, Japan; Nagoya University, Nagoya-city, Aichi, Japan; Japan Institute for Health Security, Shinjuku-ku, Tokyo, Japan

## Abstract

**Background:**

Currently, there is a global mpox outbreak. It is imperative to develop predictive diagnostic models that incorporate clinical information to effectively manage outbreak.

**Figure:**
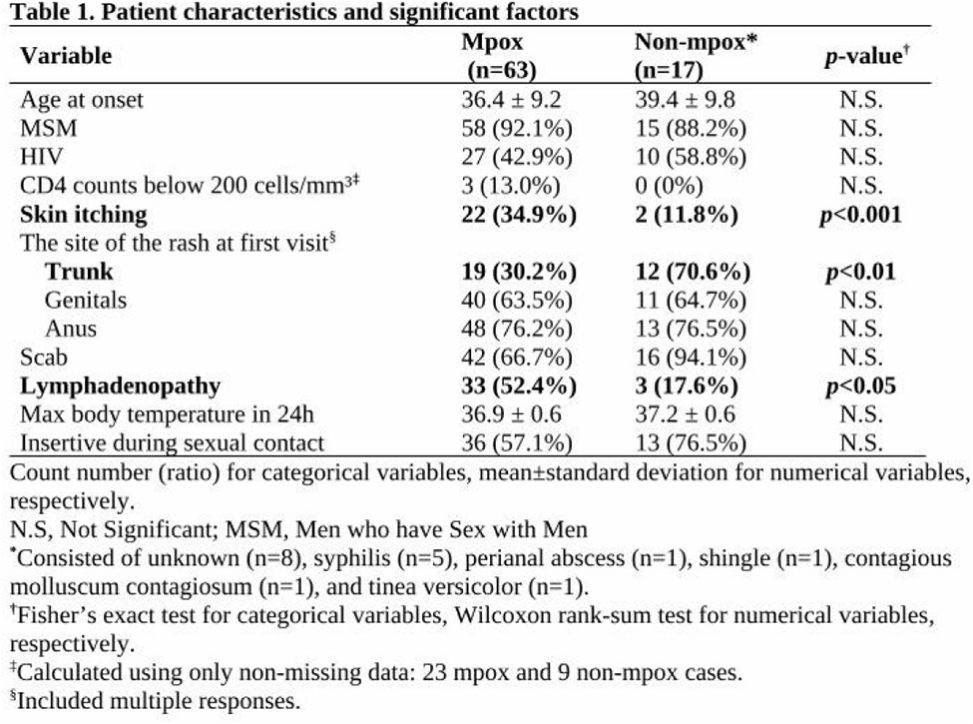

**Figure 1.**
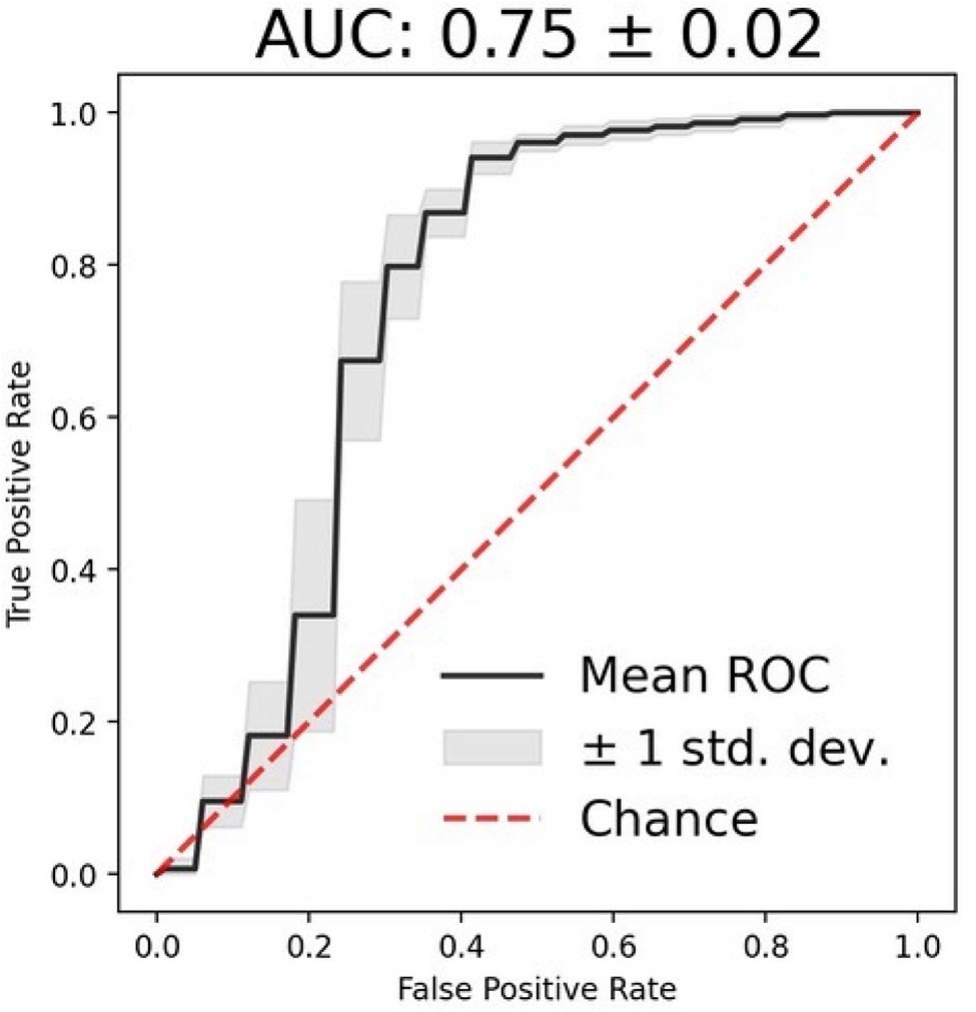
The ROC curve of lightGBM classifiers trained to predict PCR results LightGBM to predict mpox showed a sensitivity of 0.94, a precision of 0.88, a specificity of 0.56, and an area under the receiver operating characteristic curve of 0.75.

**Methods:**

This prospective cohort study was conducted at the National Center for Global Health and Medicine, a national reference center for emerging infectious diseases in Japan, from July 2022 to July 2024. The study population included patients suspected of having mpox, based on their travel and contact histories or clinical symptoms. We analyzed the data as a binary classification problem using patient demographics, medical histories, and symptoms as explanatory variables, with the PCR test results of mpox (positive or negative) as the response variable. Using a light Gradient Boosting Machine (lightGBM) with leave-one-out cross-validation, we identified significant predictive factors using Power-Full Shapley Feature Selection Method (Powershap), repeated across 100 iterations with varying seeds. The relationship among patient characteristics between positive and negative patients in the PCR results was assessed using the Mann-Whitney U and Fisher's exact tests.

**Figure 2.**
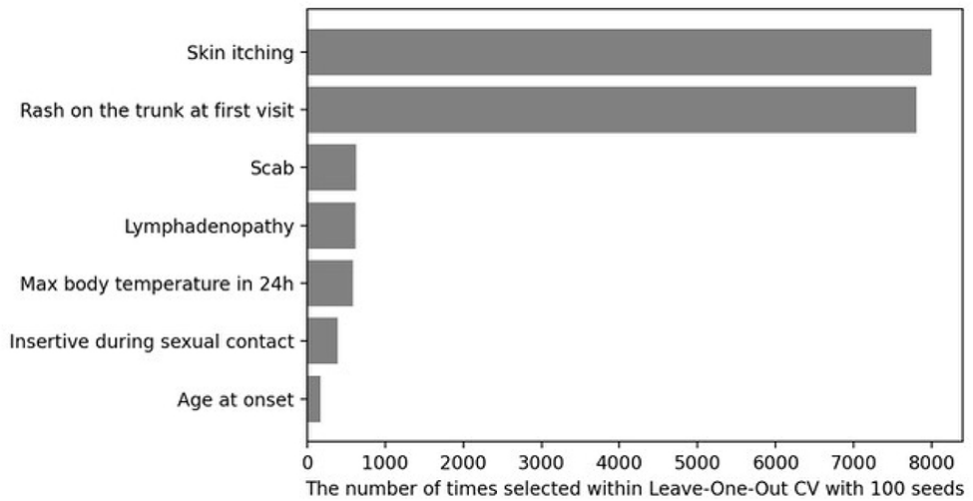
Feature importance by Powershap selection frequency in leave-one-out CV using 100 seeds Feature selection using Powershap revealed that seven of the 105 features were sufficient for prediction. Skin itching and rash on the trunk at the first visit were thought to be important clinical features in the diagnosis of mpox.

**Results:**

Of these 80 patients, 63 with mpox (clade IIb) and 17 without mpox were included (Table 1). All were male; 58 men who have sex with men were included in the mpox group and 15 in the non-mpox group, and only three mpox patients had CD4 counts < 200 cells/mm. Using lightGBM to predict mpox, we achieved a sensitivity of 0.94, a precision of 0.88, a specificity of 0.56, and an area under the receiver operating characteristic curve of 0.75 (Fig. 1). Feature selection using Powershap revealed that seven of the 105 features were sufficient for prediction (Fig. 2). Furthermore, when comparing factors between mpox and non-mpox patients, we found that skin itching and lymphadenopathy were significantly more prevalent in mpox patients, while non-mpox patients had a significant rash on the trunk at the first visit (Table 1).

**Conclusion:**

Among the clinically suspected cases of mild mpox, skin itching, lymphadenopathy, and rash on the trunk at the first visit were thought to be important clinical features in the diagnosis of mpox. Further research is needed to evaluate more appropriate predictive diagnostic models of mpox with large sample sizes and in hospitals.

**Disclosures:**

All Authors: No reported disclosures

